# Overview of the contemporary management of supracondylar humeral fractures in children

**DOI:** 10.1007/s00590-021-02932-2

**Published:** 2021-03-20

**Authors:** Sean Duffy, Oliver Flannery, Yael Gelfer, Fergal Monsell

**Affiliations:** 1 Orthopaedic Registrar, Severn Deanery, Bristol, UK; 2 Consultant Paediatric Orthopaedic Surgeon, St George’s Hospital, London, UK; 3 Consultant Paediatric Orthopaedic Surgeon, Bristol Children’s Hospital, Bristol, UK

**Keywords:** Supracondylar, Review, Treatment, Neurovascular, Cubitus Varus

## Abstract

**Purpose:**

Supracondylar fractures are common injuries accounting for approximately 15% of all fractures in children with a large body of literature on this subject.

**Methods:**

This article critically appraises the available evidence to provide an overview of the treatment options including the role and timing of surgery, the geometry of wire fixation and the management of nerve and arterial injury.

**Conclusion:**

Management decisions are based on a number of considerations particularly fracture stability. Closed reduction and percutaneous K-wire stabilisation are commonly recommended for an unstable displaced fracture. These techniques are however associated with the potential for iatrogenic neurological injury. Vascular injury is also rare but must be recognised and treated promptly to avoid significant permanent morbidity.

## Introduction

In 1959, Dr John J Gartland [1] noted “the trepidation with which men, otherwise versed in the management of trauma, approach a fresh supracondylar fracture”. Whilst the management has evolved since this description, this fracture continues to challenge contemporary orthopaedic surgeons. This article critically appraises published evidence to provide an overview of the treatment options including the role and timing of surgery, the geometry of wire fixation and the management of nerve and arterial injury.

## Epidemiology

Supracondylar fractures of the distal humerus account for approximately 15% of all paediatric fractures [2–4]. The median age of presentation is six years [5–8], and the incidence gradually reduces with age until age 15, when patients tend to present with an adult pattern [8]. This injury is reported to be more common in males [5, 8, 9] but there is a lack of consensus, some reports indicating a higher incidence in females [10, 11] and a recent evaluation of a cohort of > 63,000 children over a five year period did not demonstrate a statistically significant difference [7]. The mechanism of injury is usually a fall onto an outstretched hand, with axial transmission of body weight through the maximally extended elbow. This produces an extension type fracture, which accounts for 97–99% of injuries and may be influenced by the ligamentous laxity that is common in this age group, predisposing to elbow hyper-extension [8, 12, 13]. There is a higher incidence of injury over weekends and during the summer months [5, 7]. And falls from play equipment are frequently implicated [5, 14]. Flexion type injuries are far less common, accounting for 1–3% [5, 14], and open fractures are also rare, occurring in approximately 1% and more frequently in the older child [7].

## Anatomy and mechanism of injury

An appreciation of the morphology of the distal humerus is necessary to understand the high incidence of fractures in this region. The distal humerus is roughly triangular in the coronal plane, with a base formed by the transverse condylar masses (lateral epicondyle, capitellum, trochlear and medial epicondyle) and the sides formed by the medial and lateral supracondylar ridges. The olecranon and coronoid fossae form the centre of this triangle, with a thin area of bone proximal to the condylar masses and between the supracondylar pillars. This produces a dumbbell shape in the axial plane and the thin plate of bone acts as a stress riser that fails under excessive axial load. The relative ligamentous laxity predisposes to hyper-extension [13] and results in the olecranon acting as a fulcrum against the olecranon fossa until the anterior periosteum tears and the cortex fails. The fracture may progress to the posterior cortex with the posterior periosteum acting as a hinge, preserving stability and facilitating reduction [15].

The direction of displacement of the distal fragment may indicate whether the medial or lateral periosteum remains intact and assists in planning the appropriate reduction manoeuvre [16]. Posteromedial displacement is associated with a posterolateral periosteal tear, with preservation of the posteromedial periosteum. Placing the forearm in pronation tensions the medial periosteum and potentially assists with fracture reduction, avoiding varus malalignment. Conversely, posterolateral displacement tends to disrupt the medial periosteum and in this circumstance, supination is appropriate [17].

## Classification

A number of classification systems are used to describe supracondylar fractures and are effectively assessing stability, which is of primary importance in determining appropriate management.

It is important to initially discriminate between the common extension and unusual flexion types on the basis of the initial lateral radiograph.

**Fig. 1:**
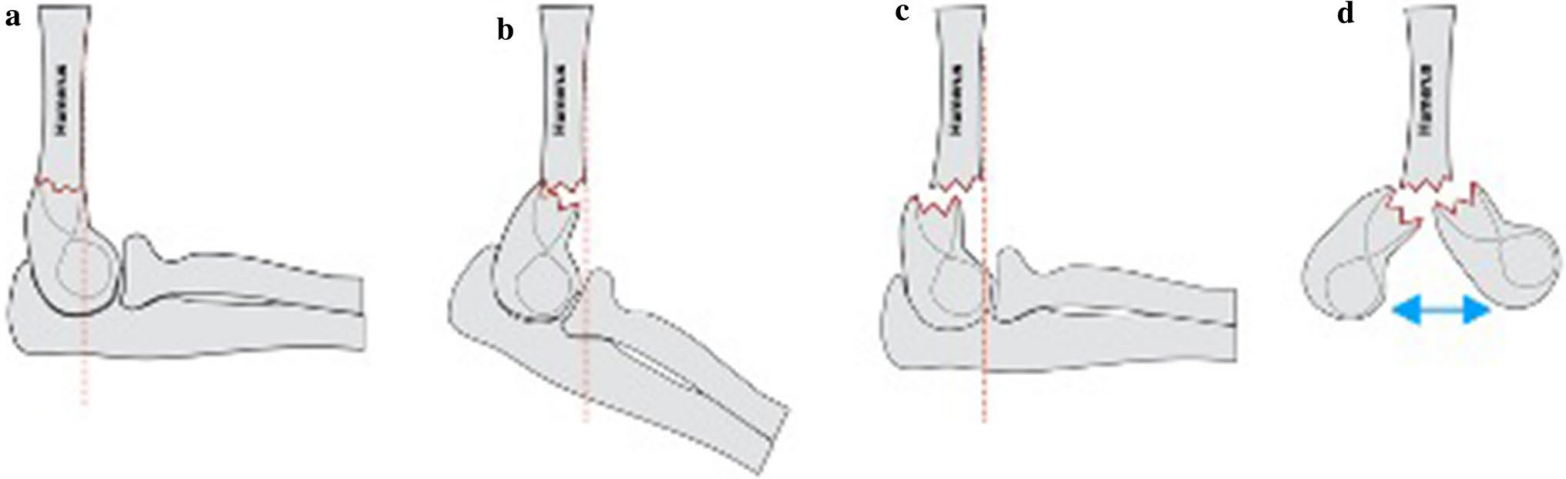
The modified Gartland classification system **a** type I non-displaced, **b** type II moderately displaced, posterior cortex remains in continuity (IIa no rotation, IIb rotation), **c** type III complete displacement and **d** type IV unstable in flexion/extension (intraoperative discovery)

Gartland [1] classified extension injuries as non-displaced, moderately displaced and severely displaced (Fig. 1). Wilkins extended this to include the concept of posterior humeral cortical contact with a rotational deformity, suggested by translation, visible in either plane [18]. Fractures were subdivided into IIa: posterior hinge intact without rotation and IIb: posterior hinge intact with rotation and this modification has good intra- and inter-observer reliability [19–22].

De Boeck [23] advocated surgical management when the medial column of the humerus was comminuted and therefore unstable, leading to alteration of Baumann’s angle. Leitch (2004) added intra-operative multiplanar instability (type IV injuries), due to complete disruption of the periosteum [24].

The AO-Muller classification system discriminates on the basis of completeness of the fracture, displacement in either AP or lateral planes and bone contact. It provides a practical guide to management and provides a reproducible method of codifying these injuries [25].

## Assessment

An accurate history and clinical assessment is crucial although not always straightforward, particularly in younger patients. Supracondylar fracture in isolation is an uncommon presentation of non-accidental injury (NAI) [26] with < 0.5% of fractures caused deliberately [27–30]. A consistent and appropriate account of the circumstances and mechanism of injury from both the patient (if applicable) and the parents are important.

Initial visual inspection considers swelling, bruising and the integrity of the skin, with any sign of bleeding suggesting an open injury. Puckering of the skin in the antecubital fossa suggests that the proximal, metaphyseal fragment has perforated the brachialis muscle and is associated with an increased risk of injury to the brachial artery and median nerve, which can also become incarcerated in the fracture site during reduction [31]. The incidence of vascular compromise is reported between 10 and 20% in displaced supracondylar fractures [16, 32]. A meta-analysis of > 5000 fractures reported an overall rate of traumatic neurapraxia of 11.3%, extension fractures with associated neurapraxia most frequently involved the anterior interosseous nerve (34.1%) and neurapraxias associated with flexion injuries most commonly involved the ulnar nerve (91.3%) [33]. Ipsilateral limb injuries, most commonly concurrent forearm fractures, occur in up to 5% [34, 35] are associated with an increased risk of neurovascular injury and compartment syndrome [36, 37].

Informal examination may be necessary in a young patient and games, particularity ‘rock, paper, scissors, ok’ can provide a rapid and accurate assessment of neurological function. Motor function in median nerve is evaluated by asking the child to make a fist (rock), radial nerve by extending the fingers (paper) and ulnar nerve by abducting the fingers (scissors). The anterior interosseous nerve is evaluated by flexing the inter-phalangeal joint of the thumb and distal inter-phalangeal joint of the index finger (OK sign). This is accompanied by an assessment of sensation, making note of sensory loss in a peripheral nerve distribution. An accurately documented neurovascular examination is essential and should be repeated before and following any intervention.

## Radiographic assessment

At birth, the distal humeral epiphysis is wholly cartilaginous, with the capitellar ossification centre appearing at age one year [38], making the diagnosis of supracondylar fracture difficult in the very young. The order of appearance and fusion of the ossific centres of the distal humerus is consistent and reliable between individuals [39] and AP and lateral plain radiographs of the elbow are usually sufficient in older children. The only radiographic sign of injury in non-displaced, stable fractures may be the presence of an effusion signified by an anterior or posterior ‘fat pad sign’, with a 75% positive prediction of an occult fracture of the elbow [40, 41].

**Fig. 2:**
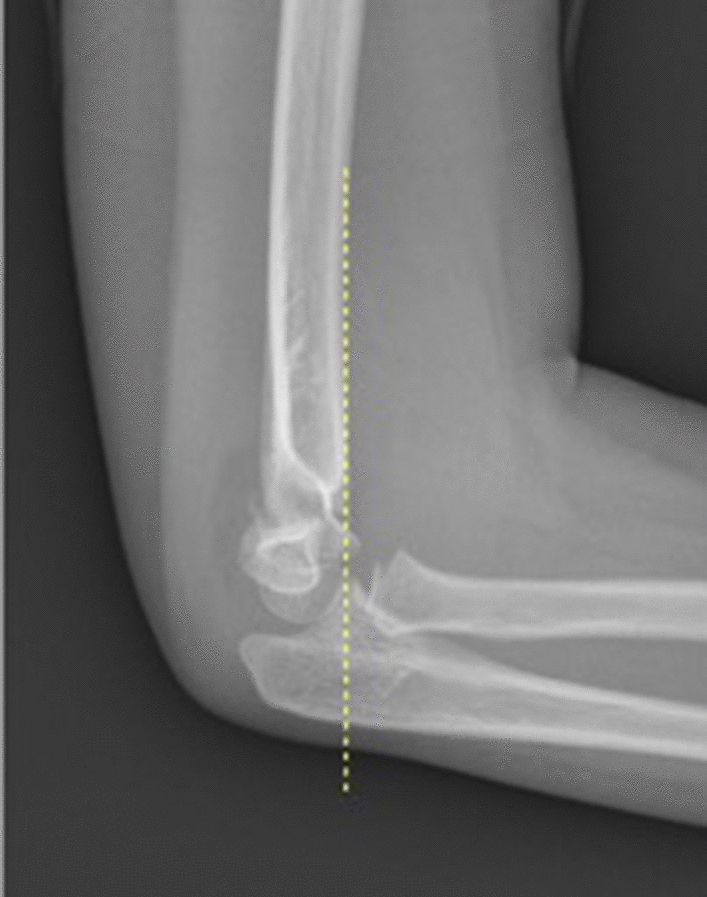
Anterior humeral line (AHL, yellow dotted line) passing in front of the capitellum suggesting posterior/extension displacement of the distal humerus

The anterior humeral line (AHL) intersects the middle third of the capitellum and is a convenient radiological sign of sagittal plane alignment (Fig. 2) [42] and an important indicator of successful reduction [43, 44].

Accurate coronal plane alignment is an important consideration and Baumann’s angle is a commonly used radiological measurement. The original description measured the intersection of a line drawn down the humeral shaft axis and a line running through the lateral condylar physis [45, 46]. The population normal is 64°–81° and angles in excess of this suggests varus angulation of the distal humerus [47] with measurement errors of up to 7° to be anticipated [48]. This measurement, whilst not identical to the carrying angle is a reasonable surrogate and can be used to predict cubitus varus deformity [49, 50].

## Specific injury patterns

### Transphyseal separation

Transphyseal separation is the least common physeal injury of the distal humerus and present in children aged under three years [51, 52]. Although rare, it is most commonly associated with birth trauma or minor falls but is also seen after deliberate injury. It should be considered as a diagnosis in the presence of elbow swelling or pseudoparalysis of the arm [53]. Plain radiographs centred on the elbow may demonstrate an abnormality of alignment of the forearm with the humeral shaft but this can be subtle, frequently overlooked and ultrasonography may be necessary to confirm the diagnosis [54]. Closed reduction with an intraoperative arthrogram is conventional and percutaneous pinning may be required in selected cases [51]. Neurovascular compromise is rare however residual cubitus varus deformity is described as a long-term consequence of this injury [51, 52, 55].

### Flexion type

**Fig. 3:**
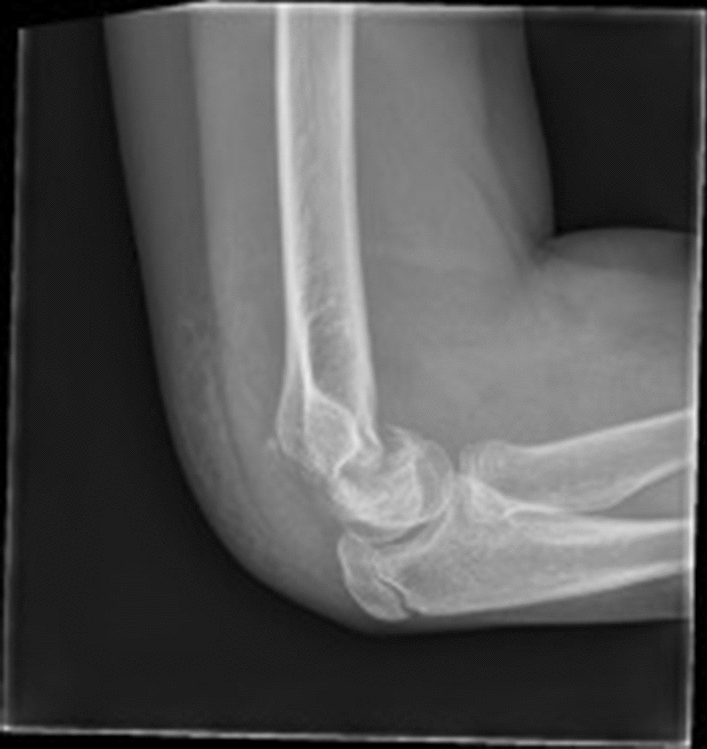
Flexion type supracondylar fracture

A flexion pattern is uncommon with rates estimated between 1 and 3% of all distal humeral fractures [38, 56]. A large population study conducted over a nine-year period reported a rate of 1.2% [12]. The mechanism of injury includes axial loading but is usually due to a direct blow to the elbow [12, 56, 57]. This results in failure of the posterior cortex and flexion of the distal fragment, which hinges on the anterior cortex/periosteum resulting in the anterior humeral line passing posterior to the capitellum (Fig. 3). This injury tends to occur in older children, is frequently displaced [12, 56], and is associated with an increased risk of ulnar nerve symptoms, which may be due to incarceration of the nerve in the fracture site, causing a block to reduction [58].

There is a lack of good quality evidence on the management of these injuries. Manipulation and casting in extension have been shown to give good results [59], although can be poorly tolerated. Closed reduction and percutaneous pinning are usually necessary but open reduction may be necessary [12, 56–58, 60].

### Extension type

There has been considerable debate about the optimum treatment of these injuries, which continues. The most controversial topics include the wiring technique, timing of surgery and the necessity to explore neurovascular structures at risk.

There is a paucity of high-level evidence to guide management with one systematic review of four randomised control trials comparing crossed wire and lateral wire pinning reporting an inability to draw a conclusion because of methodological limitations [61]. The following account attempts to present the best available evidence according to fracture type.

### Gartland I (AO 1)

This fracture pattern is inherently stable and does not require reduction, and these injuries are managed conservatively.

Liebs et al. [63] recommended a collar and cuff, as described by Blount [62] in maximum flexion (≥ 125°) in type I injuries and reported good outcomes in 327 children using Quick Disabilities of Arm, Shoulder and Hand (QDASH) and Physical Quality of Life Index (PQLI) scores. An alternative is a long-arm splint [64], with Ballal et al. [65] observing that collar and cuff in isolation provided suboptimal pain control, particularly in the immediate period following injury.

### Gartland IIa (AO 2)

The management of this fracture includes a spectrum of options that essentially depend on the assessment of intrinsic stability. Interpretation of the available literature is confounded by lack of consensus on the definition of IIa and IIb injuries [66].

Ariyawatkul et al. [67] defined type IIa as fractures with Baumann angle (BA) differing from the uninjured side by less than 5°. The authors recommended closed reduction and casting if the difference in shaft condylar angle and lateral capitello-humeral angle was < 18° compared with the uninjured side. Liebs et al. [63] also recommended a collar and cuff, often referred to as *Blount’s method* [62] in treating Gartland IIa (AO 2) injuries and reported good outcomes in 143 children using QDASH and PQLI scores. Kish et al. [68] recommended a single lateral entry pin for stabilisation of type IIa fractures.

### Gartland IIb (AO 3)

Rotational deformity is suggested radiographically by translation in either plane, the corollary is that these fracture patterns are unstable.

Closed reduction and percutaneous pinning (CRPP) is conventional treatment for IIb fractures (AO 3), as reduction with simple casting is difficult and unpredictable. Flexion > 90° is often required to maintain reduction without wire stabilisation and this potentially impairs vascular supply with an associated risk of compartment syndrome [69, 70].

Pandey et al. [71] reported a randomised control trial of MUA and casting in hyperflexion vs closed reduction and crossed K-wire pinning in type IIb and III fractures. Closed reduction and pinning were associated with superior stability, maintaining the carrying angle of the elbow.

Liebs et al. [63] reported excellent mid- and long-term health-related quality of life scores (HQoL) in 136 patients treated with CRPP and lateral K-wires for Gartland IIb fractures (AO 3).

Anatomical reduction has traditionally been recommended, in part, because of poor anticipated remodelling of the distal humerus, but also as a result of studies reporting percutaneous pinning as a safe treatment option [72, 73]. There is; however, evidence to suggest that anatomical reduction is not necessary for a good outcome [74, 75].

### Gartland III (AO 4)

Muccioli 2017 reported the use of closed reduction and collar and cuff immobilisation in hyperflexion (*Blount’s method*) in treating Gartland III fractures in the absence of neurovascular compromise and instability (in 120° of flexion) and reported very good to good outcomes in all cases [62, 76].

Liebs et al. [63] reported excellent mid- and long-term HQoL scores in 141/155 AO 4 (Gartland III) treated with CRPP with lateral 1.6 mm K wires and 14/155 with a lateral external fixator. Prashant et al. [77] compared lateral entry with crossed wires in a randomised trial (*n* = 62) and did not demonstrate a significant difference in loss of reduction, restoration of carrying angle, Baumanns angle and range of movement.

## Technique

### Wire diameter

Larger diameter wires offer increased stability, in particular in the sagittal plane [61, 78–80] without increased risk of iatrogenic ulnar nerve injury [78, 80]. The British Orthopaedic Association Standards for Trauma (BOAST) supports using 2 mm wire diameter, to improve stability [81].

### Crossed wires verses Lateral-only wires

**Fig. 4:**
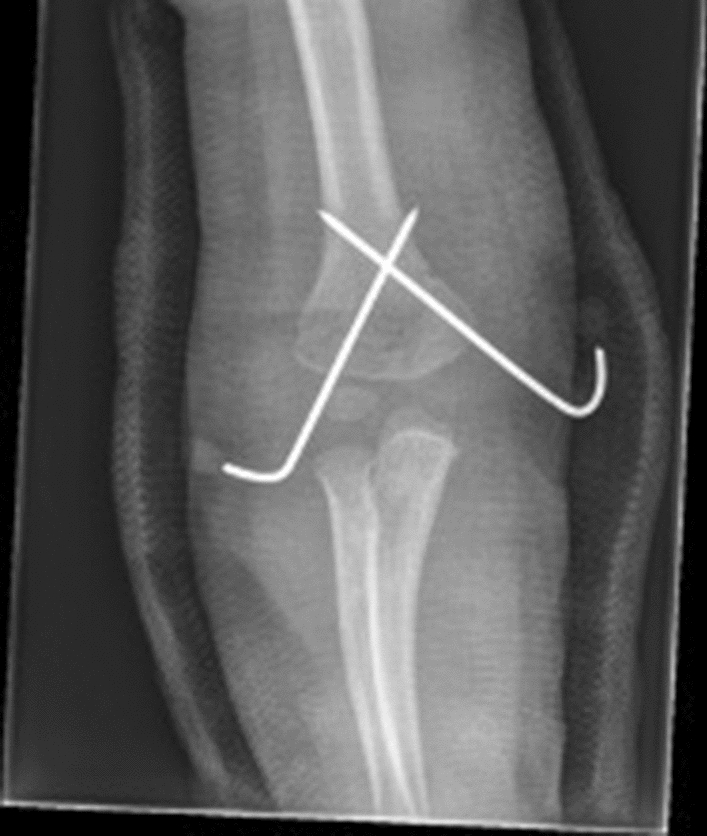
Crossed wires used to stabilise a high medial (reverse oblique) fracture pattern

Following reduction, the specific wire configuration is determined by the fracture configuration (Fig. 4), surgeon experience and personal preference.

Superior biomechanical properties have been demonstrated with crossed wires in particular in resisting rotational stresses [82–84] but it is not clear whether this improved stability is of clinical relevance [77]. Brauer et al. performed a systematic review of 35 studies comparing lateral and crossed wire techniques. Crossed wires provided improved stability (RR 0.58), however pooling prospective data alone found no statistical significance, suggesting that whilst some retrospective studies observed a difference in stability, studies with more robust methodology fail to prove clinical relevance [77, 83]. Abdel et al. [82] reported the results of a randomised study between crossed wires and lateral wires that favoured crossed wires, with no reported complications. The authors reported instability following parallel lateral wires, limiting the value of this study, as this configuration has shown inferior stability when compared to divergent wires [84]. All procedures were performed by junior trainees and the wire diameter ranged from 1.6 to 2 mm, with smaller diameter wires used in the younger children. The authors did not specify if there was an observed association between wire diameter and outcome [82].

Crossed wires are associated with iatrogenic ulnar nerve injury, with variable reported incidence. Brauer et al. [83] reported a greater than 5 times higher risk of iatrogenic ulnar nerve injury following crossed wires compared to lateral-only wires (RR 5.04). Woratanarat et al. [85] performed a meta-analysis of 18 studies involving 1315 patients and identified a 4.5 times greater risk of iatrogenic ulnar nerve injury in the crossed wires group (RR 4.5) concluding “for every 100 children treated by cross-pinning vs lateral pinning, two extra cases of loss of fixation are prevented but five extra cases of ulnar nerve damage are caused. Hence, the net effect favours lateral pinning”, where medial wires are required, meticulous technique and avoiding multiple passes of the wires are recommended.

When lateral wires are used, inserting a third lateral wire can increase stability and obviate inserting a medial–lateral wire, minimising the risk of iatrogenic injury to the ulna nerve [86, 87]. It has been established in biomechanical [84] and clinical studies [86] that divergent wire configuration creates a more stable construct than the parallel configuration.

## Complications

### Neurological injury

**Fig. 5:**
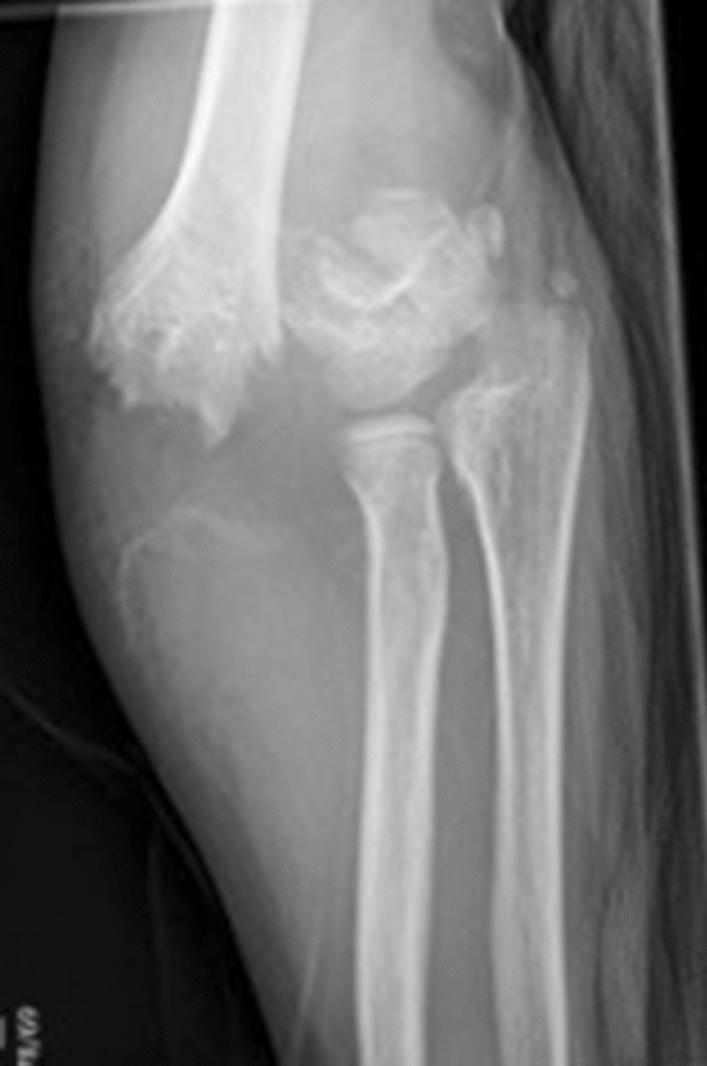
Gartland III (AO 4) injury with posteromedial displacement

Traumatic neurapraxia is regularly associated with supracondylar fractures, with a reported rate of 11.3% [33]. The anterior interosseus nerve (AIN) is most commonly injured however incidence varies with injury type. The median nerve, and in particularly, the AIN is most frequently injured following extension-type fractures (34.1% of associated neuropathies), whilst the ulnar nerve is more commonly associated with the less common flexion-type fractures (91.3% of associated neuropathies) [33, 88–91] and is likely to be related to the direction of distal humeral fragment displacement. McGraw et al. [89] identified an association between posterolateral displacement and median nerve injury and posteromedial displacement (Fig. 5) with an equal incidence of radial, median and ulnar nerve injury.

The majority of neuropathies resolve expectantly [91–94]. But ulnar nerve recovery may be less reliable [94]. It is also important to consider entrapment within the fracture site as a potential cause of neurovascular injury and may require surgical release [91, 93, 95].

Cramer et al. reported 15 median and anterior interosseus nerve injuries in a review of 101 supracondylar fractures, 13 (80%) were identified preoperatively and all fully recovered. Exploration was however necessary in three cases, two with absent pulses for median nerve/brachial artery entrapment at the fracture site [93]. These findings highlight the value of the clinical examination in identifying preoperative, injury-associated neurovascular injury.

Iatrogenic nerve injury may occur after closed manipulation, percutaneous fixation or during open procedures for reduction and vascular exploration [92]. Babal et al. [33] reported a meta-analysis, which included 5154 fractures and identified a substantial risk of injury to the ulnar nerve with medial wires and although lateral-only wires have a significantly lower risk of injury to the ulnar nerve, this technique is not risk-free and is associated with median nerve injury. Prashant et al. [77] reported a rate of iatrogenic ulnar nerve palsy of 6% with medial pinning and none with lateral pinning. Brown et al. [96] reported a review of 162 fractures with four cases of direct nerve injury and Blakey et al. [95] observed two ulnar nerves transected by wires in a review of 56 nerve injuries. Lyons et al. reported good results associated with iatrogenic ulnar nerve injuries following crossed K-wire fixation irrespective of whether the wire was removed, the nerve explored or treated conservatively [96, 97].

Ramachandran et al. reported a series of 37 radial, median and ulnar nerve injuries that presented at an average of 7.7 months after supracondylar fracture. Spontaneous recovery was noted in 27 at an average time of 7–8 months and 81% had an excellent outcome. Exploration was necessary in 10 patients, demonstrating nine nerves in continuity and one transection. Ulnar neurolysis was necessary in six for entrapment within a fibrous/callous cubital tunnel and four required nerve grafting [92].

### Vascular injury

Blakey et al. reported the long-term follow-up of 26 children with a “pink and pulseless” hand in whom 23 developed a contracture, with deformity of the forearm and hand. One patient had undergone exploration of the vessel at an interval of 48 h but three patients, who underwent urgent exploration, did not develop a contracture. Late exploration was performed in 21 cases and identified entrapment of the vessel within the fracture site in nine and constriction by scar tissue in 12, decompression returned pulsatile flow in all cases [95].

Vascular injury noted at the time of presentation requires urgent fracture reduction. Absence of a palpable radial pulse in an otherwise perfused (‘pink and warm’) hand usually resolves after reduction of the fracture and rarely requires surgical exploration [77]. Exploration, where required, should be performed by surgeons with the ability to perform small vessel repair [81] and a tourniquet should not be routinely used [95].

Several studies have reported an association between absent radial pulse and median nerve injury and this combination of clinical findings should alert to the possibility of neurovascular bundle injury/entrapment at the fracture site [98, 99].

Ischemic contracture affects primarily the flexor compartment due to prolonged muscle ischaemia but several studies have reported a low incidence of contracture, in the absence of concomitant neurological injury [100–102].

Mangat et al. reported the outcome of 19 patients presenting with a perfused pulseless hand of whom 11 were treated conservatively after closed reduction. Delayed exploration was required in four, of which three had median and/or anterior interosseus nerve palsy at presentation. Urgent exploration was performed in eight patients and the brachial artery was tethered at the fracture site in six [99].

**Fig. 6:**
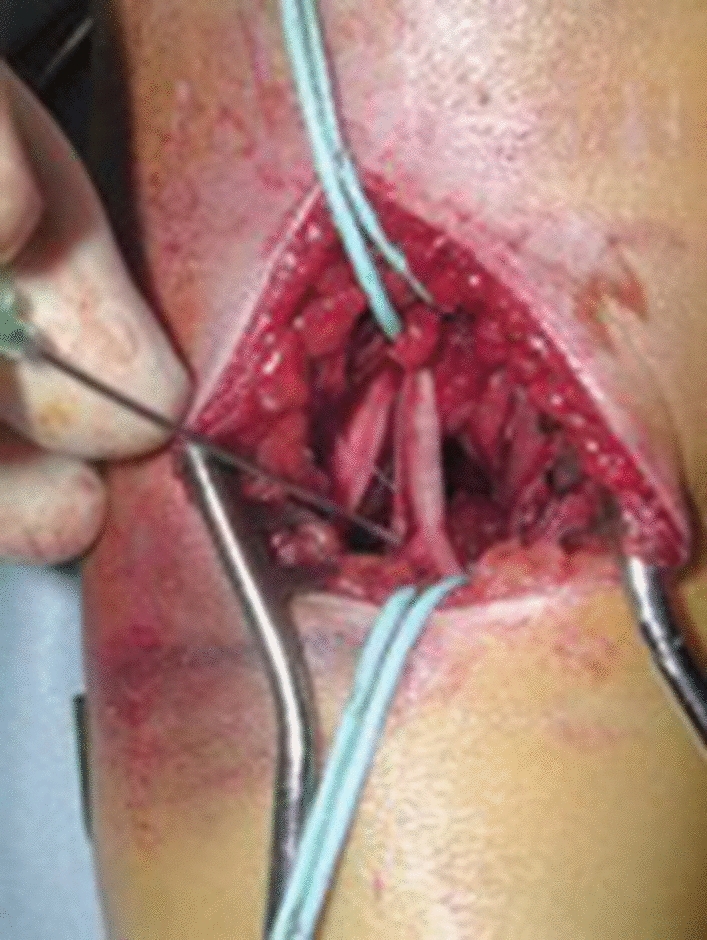
Exploration of the brachial artery and median nerve for neurovascular injury

**Fig. 7:**
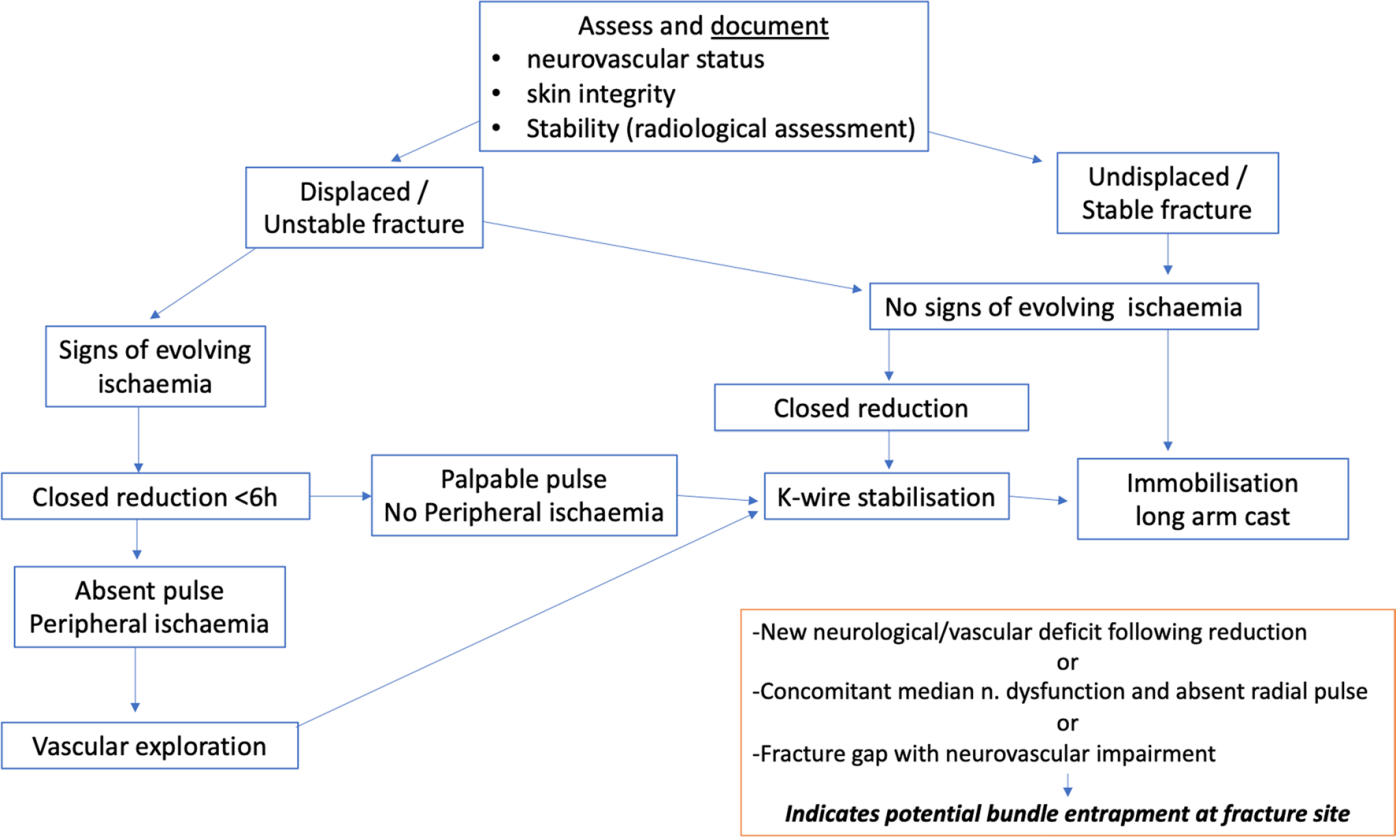
Management flowchart for supracondylar fractures related to adequate and inadequate perfusion

Rasool et al. [103] recommended exploration prior to manipulation of postero-laterally displaced supracondylar fractures with an absent or weak pulse and median nerve symptoms where clinical signs of brachialis buttonholing exist, due to the proximity of the neurovascular bundle (Fig. 6). The authors reported the results of exploration in 27 patients, with signs of median nerve injury in 22 patients at presentation and the neurovascular bundle was noted to be in immediate proximity to, tethered or interposed within the fracture site in all cases (Fig. 7).

### Cubitus varus

Conventional wisdom considers a varus elbow deformity as a cosmetic problem but recent studies have identified associated functional disadvantage. Posterolateral rotatory instability (PLRI), snapping triceps, progressive varus deformity of the ulna and elbow joint malalignment have been described [104, 105], often presenting decades after the onset of the deformity [105].

Alteration of the normal mechanical axis increases the tensile force through the lateral structures and medial displacement of the triceps creates a supination force on the olecranon [105]. O’Driscoll et al. [105] described lateral ulnar collateral ligament attenuation in patients with untreated postero-lateral rotatory instability with radial head dislocation in severe cases.

Lateral closing wedge osteotomy of the humerus is an effective treatment with a reliable outcome [106]. Alternative techniques have also been described including a step-cut osteotomy [107, 108], dome osteotomy [109], distraction external fixator [110] and computer-aided osteotomies [111].

### Cubitus valgus

Valgus deformity is a rare complication of supracondylar fractures with a reported incidence < 1–3% [112, 113] and is more frequently encountered following malunion/physeal arrest in lateral condyle fractures. Similarly to varus correction, various surgical techniques have been reported for post-traumatic cubitus valgus including Ilizarov frame [114], dome osteotomy [115] and step-cut osteotomy [116].

## Final remarks

Supracondylar fractures that represent approximately 15% of all paediatric fractures [2–4] are associated with a 10–20% rate of vascular compromise in displaced fractures [16, 32] and > 11% incidence of traumatic neurapraxia in all fractures [33].

Fracture management depends on stability, with closed reduction and percutaneous pinning being the preferred treatment for the unstable displaced fracture [63, 73, 77].

Nerve injuries are related to injury subtype, with posterolateral displacement associated with ulnar nerve injury, posteromedial displacement associated with an equal incidence of radial, median and ulnar nerve injury [89] and flexion-type fractures with injury to the ulnar nerve [33, 89, 91].

The vast majority of neuropathies resolve expectantly [91–94]. Anterior interosseous nerve palsy is the most common neuropathy and is most commonly caused by traction either at the time of injury or during manipulation [33].

Median nerve symptoms that exist with vascular compromise suggest neurovascular bundle entrapment in the fracture site [98, 99] with improved outcomes associated with early exploration [95, 99].

Current evidence is generally poor and limited by inadequate methodology with low-quality retrospective studies and small population numbers. These do little to inform the continuing debate and there is a need for multi-centre prospective trials to outline the optimum management for these injuries.
